# Hierarchical Coordination and Institutional Improvement of China’s Laboratory Animal Welfare Standard System

**DOI:** 10.3390/ani16152301

**Published:** 2026-07-24

**Authors:** Wenlong Zhao, Baolong Li

**Affiliations:** Drug Safety Evaluation Center, Heilongjiang University of Chinese Medicine, Harbin 150006, China; 18846765451@163.com

**Keywords:** laboratory animal welfare, standard, 3R principle, welfare and ethics review

## Abstract

Ecological civilization refers to the harmonious development of humanity and nature, with its core essence lying in respecting nature and rejecting “anthropocentrism”—a concept that aligns perfectly with the principles of animal protection. The protection of laboratory animals, which are most closely tied to human scientific progress, is particularly crucial. For centuries, laboratory animals have served as substitutes and models for humans, treated merely as tools. With the advancement of humanitarianism, however, the protection of laboratory animal welfare has gradually gained greater attention. China has established a system for protecting the welfare of laboratory animals through foundational laws and tiered standards, translating internationally recognized animal protection concepts—such as the “3Rs” principle and the Five Freedoms—into actionable operational requirements, thereby implementing concrete measures to safeguard the welfare of laboratory animals. However, China’s relevant standards still face issues such as insufficient enforcement and a lack of mandatory provisions. To safeguard the welfare and rights of laboratory animals, China must integrate animal welfare into the development of an ecological civilization, improve laws and regulations, strengthen talent development, promote technological innovation, and establish a comprehensive system of standards to ensure the welfare of laboratory animals.

## Simple Summary

Laboratory animals are vital to scientific research, yet their welfare has long aroused widespread concern. This review outlines China’s evolving legal and standard system for laboratory animal protection, covering early quality control rules and updated guidelines focusing on animals’ physical and psychological well-being. It compares China’s regulatory framework with those of the EU and the US, summarizing domestic progress and persistent hurdles. Major challenges include low compliance with voluntary standards, insufficient training for researchers, occupational burnout of animal technicians, and inadequate public transparency of animal experiments. Corresponding solutions are put forward: stricter laws, systematic vocational training, improved housing and feeding facilities, and more public disclosure of experimental animal treatment. Although China has built a relatively complete welfare system, gaps still exist between written standards and real-world practice. This review offers practical references for policymakers, researchers and animal caretakers to boost laboratory animal welfare at home and worldwide.

## 1. Introduction

### 1.1. Historical Background of Laboratory Animals

Most laboratory animals are animals that have been artificially reared, with their microbial and parasitic loads controlled, and whose genetic background or origin is clearly defined; they are used for scientific research, teaching, production, testing, and other scientific experiments [1]. As the foundation of scientific research, laboratory animals have a long history of development. As early as the Renaissance, there were precedents for using animals to replace humans in experiments; however, the animals of that era were far from what we would today call laboratory animals. Today, with the successive emergence of inbred strains, disease model animals, and genetically engineered animals, the field of laboratory animals is evolving toward standardization and regulation.

With the progress of the times and evolving philosophies, as surrogates for humans, there is a growing emphasis on ensuring that animals’ physiological and psychological needs are met—not only to guarantee the success of scientific research but also to safeguard the rights of animals during the experimental process [2]. In 1938, to mitigate the risks posed by drugs with unknown side effects to humans, the U.S. Food and Drug Administration (FDA) required that drugs be tested on at least two species of animals before being used in human clinical trials; Today, the application of artificial intelligence systems, machine learning models, organoids, organ-on-a-chip technologies, and other complex cell culture methods has significantly reduced the use of laboratory animals. However, countries in Asia, Europe, and elsewhere still insist on requiring drug testing on animals [3]. Laboratory animals remain irreplaceable in scientific research, making the protection of their welfare particularly important and urgent [4].

Animal welfare refers to meeting the basic physiological needs of animals, enabling them to live in comfort and health and to die without suffering—that is, treating living animals with kindness and minimizing the pain associated with their death [5]. While ensuring animal health and life, it is also key to the accuracy and ethical integrity of scientific experiments. Since animals cannot directly communicate their experiences, the evaluation tools used to measure whether “optimization” has been achieved must undergo empirical validation. This is the core value of “construct validity”—it ensures that the indicators we use to assess an animal’s condition accurately reflect its intrinsic welfare level [6]. Once scientifically reliable assessment tools are in place, the protection of laboratory animal welfare must be further regulated by higher-level codes of conduct. As early as 1959, zoologist William Russell and microbiologist Rex Birch systematically proposed the “3R Principles”—Reduction, Replacement, and Refinement—in *The Principles of Humane Experimental Technique* to protect the rights of laboratory animals. To this day, these principles remain internationally recognized by the academic community as the highest guiding standards for laboratory animal welfare [7]. The Five Freedoms, established in 1974, define the rights to which animals are entitled: (1) freedom from hunger, thirst, and malnutrition; (2) freedom from discomfort; (3) freedom from pain, injury, and disease; (4) freedom to express normal behavior; and (5) freedom from fear and distress. They systematically define the basic welfare conditions to which animals are entitled across five dimensions: physiological, environmental, health-related, behavioral, and psychological [8]. In 2020, Tom L. Beauchamp and David DeGrazia proposed six ethical principles for animal experimentation, based on the 3Rs, that address both societal benefits and animal welfare. The principles regarding social benefits are the following: (1) No Alternative Method; (2) Expected Net Benefit; and (3) Sufficient Value to Justify Harm. The principles regarding animal welfare are the following: (1) No Unnecessary Harm; (2) Basic Needs; and (3) Upper Limits to Harm. These principles are not intended to replace the 3Rs, but rather to supplement and deepen their core ideas, reflecting a higher-level and more comprehensive ethical consideration of animal welfare [9].

### 1.2. The Development of International Laboratory Animal Welfare

Ensuring the quality of animal welfare in research on a global scale is crucial to international scientific collaboration. The significant differences in animal welfare across countries stem primarily from each nation’s unique cultural traditions and are influenced by various factors, including economic development and religious ethics. In certain countries or religious and cultural contexts, where anthropocentric ethical views prevail, animals are viewed merely as exploitable resources; Under this perspective, the protection of animal welfare stems primarily from a recognition of moral obligations toward humans. Conversely, in other cultures (such as Hinduism), the continuity of life and the principle of nonviolence are emphasized, and the humane treatment of animals is viewed as a practice related to one’s own karma and spiritual cultivation. Therefore, in international scientific collaboration, it is essential to first establish a minimum consensus on animal welfare standards among all parties and to effectively translate these into concrete actions [10].

#### 1.2.1. Development of Laboratory Animal Welfare in the European Union

As a leading frontier in life sciences research, the European Union has established the world’s most influential standards for laboratory animal welfare through continuous revisions to laws and regulations and the strengthening of regulatory systems. The 1997 Treaty of Amsterdam and the 2007 Treaty of Lisbon defined animals as “sentient beings”, calling for the enhancement of animal protection to achieve animal welfare. These treaties established animal welfare provisions across a broad range of sectors, primarily focusing on agriculture, transport, the internal market, and research, with supplementary provisions covering fisheries, technological development, and space [11,12].

In September 2010, the European Union adopted the Directive of the European Parliament and of the Council on the protection of animals used for scientific purposes (2010/63/EU) [13]. First, the Directive explicitly recognizes the intrinsic value of animals, legally establishing their status as distinct from other goods and property. Second, the Directive fully incorporates the “3Rs Principle” into the regulatory framework, requiring Member States to actively implement it in animal experimentation. Third, the Directive expands the scope of protection, covering not only basic research and education but also extending protection to the last third of vertebrate gestation and to cephalopods. Fourth, it imposes strict restrictions on experiments involving nonhuman primates and, in principle, prohibits the use of great apes in experiments. Furthermore, the Directive mandates the establishment of a continuous ethical review system to enhance the transparency and enforceability of the regulations [14]. Meanwhile, in 2014, the United Kingdom announced plans to reduce the use of animal testing in scientific research, with a focus on replacing such tests with “scientifically valid alternatives” wherever possible [15]. More recently, in 2021, the European Parliament passed a resolution by an overwhelming majority calling on the European Commission to develop an action plan to phase out animal testing [16].

#### 1.2.2. Development of Laboratory Animal Welfare in the United States

Historically, in 1966, the United States enacted the Laboratory Animal Welfare Act, which was originally intended to safeguard pets and ensure the humane care and treatment of research animals; in 1970, it was renamed the Animal Welfare Act, expanding the scope of animal protection to include all warm-blooded animals and introducing civil penalties for violations. In 1990, it was renamed the “Pet Protection Act”; however, a drawback of this legislation was that it excluded birds, rats, and mice bred for research purposes from its scope of protection [17]. Although animal protection organizations filed multiple lawsuits attempting to overturn this provision (such as the 1991 case of Animal Legal Defense Fund v. USDA), the U.S. Congress ultimately passed an amendment in 2002 that permanently confirmed the exemption status of these three categories of animals at the legislative level. Meanwhile, the Public Health Service Policy on Humane Care and Use of Laboratory Animals, implemented by the Office of Laboratory Animal Welfare under the Health Research Extension Act of 1985, covers all vertebrates used in institutions receiving PHS funding (including rats, mice, and birds exempted under the Animal Welfare Act). It requires institutions to follow the Guidelines for the Care and Use of Laboratory Animals, which stipulate requirements regarding research personnel, facilities, and the housing and care of laboratory animals [18].

Recently, in December 2022, the U.S. Congress passed the FDA Modernization Act 2.0, formally repealing the mandatory requirement—in place since 1938—that preclinical trials for new drugs must use animals. The Act explicitly permits the use of “nonclinical testing” methods, including cell-based assays, organ-on-a-chip technologies, and computer models. This change marks a shift in animal welfare from a focus solely on “how to care for animals” to a focus on “whether animals are necessary.” In 2025, the U.S. Food and Drug Administration (FDA) released a roadmap proposing that animal testing should shift from being the “norm” to the “exception”, with plans to phase out routine animal testing [19].

Although the European Union and the United States have taken different paths in developing regulations on laboratory animal welfare, their core frameworks are highly similar: all have established review systems based on institutional ethics committees and have incorporated the “3Rs” into their mandatory policy frameworks. The European Union and the United Kingdom tend to regulate through directly binding directives or domestic laws; countries such as the United States, Canada, and Australia, on the other hand, rely more on a combination of federal policy guidelines and national accreditation systems like AAALAC, exhibiting a “soft but effective” regulatory approach. Despite these differences, a clear international trend has emerged: a shift from “optimizing animal care” to “reducing and even replacing the use of animals”, which provides an important reference for the refinement of China’s standards system.

### 1.3. Historical Development of Laboratory Animal Welfare in China

#### 1.3.1. Unique Characteristics of Laboratory Animal Welfare in China

Faced with the historic task of economic and technological development in modern China, the country adopted a policy of “focusing on economic development.” At a stage when the level of economic development and public awareness were not yet fully mature, animal welfare—as a nonproductive issue—was long relegated to the margins of the policy agenda. It was not until the economy and society had reached a more advanced stage of development and public awareness had gradually increased that animal welfare issues gradually entered the institutional policy framework [20]. At the same time, as international standards for animal welfare became increasingly stringent, China initially lacked corresponding regulations and standards, and this gap had a substantial impact across multiple sectors. Because China had not yet established strict regulations at that time, exports of certain traditional Chinese medicines and agricultural products were blocked due to noncompliance with internationally recognized animal welfare standards, directly affecting pharmaceutical trade and scientific research collaboration. Furthermore, in transnational clinical drug trials and joint research and development projects, foreign partners often required Chinese counterparts to provide experimental conditions that met international animal welfare standards. This “hard constraint” objectively prompted China to incorporate laboratory animal welfare into its institutional agenda during its early stages of development [21].

The legalization process of laboratory animal welfare in China can be traced back to the promulgation of the “Regulations on the Management of Laboratory Animals” (hereinafter referred to as the “Regulations on the Management”) in 1988. These regulations not only legally defined laboratory animals but also, for the first time, established mandatory requirements for animal protection.

However, unlike in Europe and the United States, where the system relies primarily on specialized legislation and mandatory enforcement, China’s modern animal welfare framework has taken a unique path: it is achieved not only through laws and regulations but also, to a greater extent, through mandatory or recommended standards. This technical regulatory system, centered on “standards”, complements—yet differs from—the regulatory model in Europe and the United States, which is centered on “law.” Standards are more closely aligned with practical operations and can flexibly adapt to technological advancements; however, they also face challenges such as lower legal standing and insufficient enforcement capacity.

#### 1.3.2. Animal Welfare Standards System Centered on the Five-Tier Model

Standards represent the unified technical requirements needed in fields such as agriculture, industry, services, and social welfare, and serve as best practices across various sectors [22]. As early as 1958, the Standards Bureau of the State Science and Technology Commission issued the “Interim Measures for Drafting National Standards”, which clearly outlined the requirements for drafting standards and included guidelines on commonly used symbols and numerical notation; although these were merely “interim measures”, they still possessed regulatory authority, marking the beginning of China’s standardization efforts moving toward legalization and standardization. In 1988, the state promulgated the “Standardization Law of the People’s Republic of China” (hereinafter referred to as the “Standardization Law”), which formally defined the concept of ‘standards’ for the first time to regulate industry order. The 2017 revision of the “Standardization Law” further expanded the scope of application of standards from the industrial sector to various sectors such as agriculture, services, and healthcare, establishing the core status of standards at the technical level [23]. To implement this law, various “Implementation Regulations” were issued, detailing the specific requirements for standard implementation that regions, industries, and departments must follow [24]. The 2017 Standardization Law established a comprehensive standard system comprising five tiers: national standards, local standards, industry standards, group standards, and enterprise standards, each serving as the highest technical requirement at different levels and in different fields.

Animal welfare standards exist across these five tiers, serving as technical specifications at different levels and in different fields. The development of animal welfare in China lagged behind that of Western developed countries. In the early days, animal experiments were conducted solely to achieve experimental results, with animals treated humanely only as a means to that end. For example, the “Regulations on the Management” merely mentioned requirements such as “caring for animals” and “preventing distress”, These requirements are closer to operational standards for animal management than to animal-centered welfare safeguards. The ambiguity of this wording reflects the characteristics of China’s laboratory animal welfare regulations during their early stages. In practice, these provisions are viewed more as management guidelines than as institutional commitments to animal welfare [25].

In 1994, building upon the “Regulations on the Management”, China issued standards such as GB 14924-1994 “Complete Nutritional Feed for Laboratory Animals” and GB 14925-2023 “Laboratory Animals: Environment and Facilities”, thereby establishing the first systematic standard framework for laboratory animals [26,27]. However, the primary purpose of these standards remained quality control; specific requirements—such as controlling laboratory temperature, humidity, housing conditions, and feed—were intended to safeguard animal health and prevent interference with experimental results. Although there are no explicit provisions guaranteeing animal welfare, the specific requirements stipulated in the aforementioned standards—such as feed composition—are intended to prevent feed from causing variable effects on animal experiments. However, these requirements also provide optimal living conditions for the animals and meet their basic survival needs, thereby passively and indirectly achieving the welfare of laboratory animals [28].

In 2006, the issuance of the “Guiding Opinions on the Humane Treatment of Laboratory Animals” (hereinafter referred to as the “Guiding Opinions”) formally established, in the form of an independent document, the humanitarian requirements for the humane treatment of animals and the assurance of their welfare; For the first time, the “3Rs” principle was introduced and required to be followed in experiments. Specific welfare standards were established for procedures such as animal housing, transportation, handling, and euthanasia, and regulations compliant with international standards were formulated, bringing China’s laboratory animal management in line with international practices [29]. When revising standards such as the “Environmental and Facility Requirements for Laboratory Animals”, the state incorporated provisions related to laboratory animal welfare, transforming recommended guidelines into mandatory requirements and shifting the protection of animal welfare from a voluntary practice to a legal obligation. In recent years, standards directly addressing animal welfare have been continuously issued, such as the “Guidelines for Ethical Review of Laboratory Animal Welfare” and the “General Rules for Laboratory Animal Welfare”, which clearly stipulate specific requirements for ensuring the welfare of laboratory animals at the national level. Different regions, organizations, and industries have also begun to explore new standards tailored to their specific characteristics, ensuring that standard requirements are practical and easy to implement [30].

## 2. Structure of China’s Laboratory Animal Welfare Standards System

### 2.1. Overview of Pioneering Key Standards

There are currently 110 national standards and 138 industry standards related to laboratory animals. Local standards, which are designed to meet the specific requirements of different regions, are the most numerous, totaling 184; industry and enterprise standards number only 9. However, few of these standards were specifically established to ensure the welfare of laboratory animals (Table 1). As a nonindependent operational guideline, welfare considerations permeate various aspects of laboratory animal management—including genetics, microbiology, facilities, transportation, and housing—and are referenced in numerous standards. For example, the *Environmental and Facility Requirements for Laboratory Animals* specifies that cage structures, space, and bedding materials must align with the animals’ physiological characteristics and welfare requirements, and that appropriate welfare equipment must be provided [27]. However, most of the aforementioned standards are voluntary and lack direct enforceability. To address this, China has established a law enforcement mechanism centered on administrative licensing: with the approval and annual inspection of the “Laboratory Animal Production License” and “Use License” serving as the linchpin, welfare standards are set as prerequisites for obtaining and renewing these licenses; local science and technology authorities conduct routine inspections through the “double random, one public” system, linking inspection results to license validity, research project applications, and credit records; Violators face warnings, orders to shut down operations, or even license revocation under the “Regulations on the Management of Laboratory Animals”; in severe cases, they are included in the scientific research misconduct registry and barred from applying for projects for one to five years. Through this three-stage closed-loop system of “licensing and market access—process supervision—credit-based sanctions”, the nonmandatory standards are thereby institutionalized and given binding legal force [31].

Based on the distribution characteristics of the aforementioned standard system, the following standards establish specific provisions for laboratory animal welfare from various perspectives, forming the technical foundation for current laboratory animal welfare management in China.

### 2.2. National Standards

#### 2.2.1. Laboratory Animal—Guideline for Ethical Review of Animal Welfare GB/T 35892-2018

The “Laboratory animal—Guideline for ethical review of animal welfare” (hereinafter referred to as the “Review Guidelines”), as the earliest existing national standard related to laboratory animal welfare, was introduced in 2018 based on standards for laboratory animal microbiology, parasitology, feed, environment, and facilities, and in accordance with the provisions of the “Guiding Opinions.” It is a recommended national standard; It provides standardized principles and guidelines for the review work of laboratory animal ethics committees, stipulating that all teaching, research, and production activities involving laboratory animals must undergo welfare and ethics review.

The “Review Guidelines” provide new definitions for laboratory animals, animal welfare, and animal ethics, specifying that welfare is not merely about ensuring the animals’ life and health, avoiding pain, and providing adequate external conditions; the animals’ right to live happily is also of paramount importance. The “Five Freedoms” principle has been formally incorporated into the standard. This ensures that animals live in a comfortable, healthy, and happy natural state. Regarding animal euthanasia, the Review Guidelines establish the concept of a “humane endpoint”: upon learning the experimental results, the experiment should be terminated promptly before the animal experiences pain or at an early stage of pain. Through reasonable judgment and the implementation of a humane endpoint, pain can be prevented or alleviated while achieving scientific objectives [45].

The *Review Guidelines* outline the core components of welfare and ethics reviews, setting forth detailed requirements regarding review principles, subjects of review, review timelines, animal sources, experimental protocols, and technical operating procedures for veterinarians. By overseeing and reviewing specific operational steps, the guidelines ensure that the stipulated standards are effectively implemented [32].

#### 2.2.2. Laboratory Animals—General Code of Animal Welfare GB/T 42011-2022

In 2022, the General Administration of Market Regulation and the Standardization Administration of China jointly issued the “Laboratory animals—General code of animal welfare” (hereinafter referred to as the “General Principles”), which is the first recommended national standard explicitly titled “Welfare of Laboratory Animals.” During its development, the “General Principles” built upon previous standards and incorporated elements from the International Code of Animal Health for Terrestrial Animals to ensure alignment with international standards. Regarding fundamental concepts, the standard retains the definitions of laboratory animals and welfare established in the “Review Guidelines” and adheres to the “3Rs” principle and the Five Freedoms. The “General Principles” further specifies welfare requirements concerning environmental enrichment, housing, and feed; For the first time, it distinguishes between the concepts of suffering and pain, defining pain as: an unpleasant sensory and emotional experience associated with actual or potential tissue damage. Suffering is defined as: an unpleasant, undesirable state resulting from multiple harmful stimuli or the absence of positive stimuli. This distinction expands welfare safeguards from the mere control of physical harm to comprehensive care for psychological experiences, providing a more complete theoretical basis and practical guidance for specific procedures such as anesthesia and analgesia, environmental enrichment, and humane euthanasia [33].

As a standard established to directly safeguard the welfare of laboratory animals, the “General Principles” does not advocate for an unrestricted policy of “do no harm.” While prioritizing animal welfare, this standard introduces the concept of risk–benefit analysis. Risk–benefit analysis refers to the process of weighing the potential adverse effects of experimental procedures on animals against the potential benefits to the research project. The appropriate use of risk–benefit analysis allows for the fulfillment of welfare requirements while maximizing research benefits. The implementation of risk–benefit analysis—such as establishing reasonable humane endpoints—enables the achievement of experimental objectives while ensuring animal welfare [46].

The General Principles introduce concepts such as biosafety, biohazards, animal injury, and zoonotic diseases. These provisions not only address the welfare of laboratory animals but also assess potential risks to personnel and facilities arising from experiments, emphasizing the protection of both laboratory animals and researchers.

Although these national standards are voluntary, they have become a mandatory prerequisite for work involving laboratory animals through the review mechanisms of Institutional Animal Care and Use Committee (IACUC). The establishment of animal welfare ethics committees has become a standard institutional arrangement at most research institutions in China; their statutory responsibilities explicitly include the ethical review and process oversight of all animal experimentation protocols to ensure the scientific rigor and impartiality of the review process. In terms of the review process, the committees implement full-cycle management of experimental protocols through “pre-review—on-going supervision—post-review”: pre-review assesses the necessity of the project and compliance with the 3Rs principles; on-going supervision monitors actual implementation through regular follow-up inspections (typically no longer than six months); and upon project completion, an ethical post-review report is submitted for final review. Projects that fail to pass the review are not permitted to conduct animal experiments. Thus, the recommended standards gain substantive enforceability through regulatory references and licensing management, ensuring that welfare requirements are effectively implemented [25].

### 2.3. Local Standards

#### 2.3.1. Code of Practice for the Welfare and Ethics of Laboratory Animals DB32/T 2911-2016

The *Code of Practice for the Welfare and Ethics of Laboratory Animals* (hereinafter referred to as the *Code of Practice*) was issued by the Jiangsu Provincial Bureau of Quality and Technical Supervision in 2016, drawing upon relevant national laws and regulations as well as international conventions such as the *Helsinki Declaration*. As a recommended local standard predating the “Review Guidelines”, the “Code of Practice” is more region-specific. During its formulation, the “Code of Practice” used the “Jiangsu Province Laboratory Animal Management Measures” as a foundational reference, integrating the standard with local animal management methods to refine the details of animal welfare work and ensure the effective implementation of local management regulations. The “Code of Practice” provided practical experience for the issuance of national standards, revealed shortcomings in welfare implementation, and effectively promoted the introduction of national standards [36].

#### 2.3.2. Technical Specifications for Ethical Review of Laboratory Animal Welfare DB11/T 1734-2020

In 2020, the Beijing Municipal Market Supervision Administration issued the “Technical Specifications for Ethical Review of Laboratory Animal Welfare” (hereinafter referred to as the “Review Specifications”), marking the first specialized technical specification for ethical review of laboratory animal welfare issued by a municipality directly under the central government. The “Review Specifications” focus on the technical aspects of the review process, providing more detailed provisions regarding the composition of ethics committees, time limits for review procedures, and criteria for determining review conclusions. The specifications explicitly require ethics committees to conduct item-by-item assessments of the implementation of the “3Rs” principles in experimental protocols and establish a re-review and appeal mechanism to ensure the fairness and transparency of the review process [37].

Differences in local standards are primarily reflected in aspects such as the characteristics of animal breeds and cage specifications. For example, Jiangsu Province, as a major production base for cages, took the lead as early as 2006 in issuing local standards such as “Laboratory Animal Cages and Equipment: Metal Cages”, which specify in detail the minimum floor area and cage height required for different animals, including mice, rats, and rabbits. Provinces such as Sichuan and Hunan, on the other hand, have placed greater emphasis on developing laboratory animal resources with regional characteristics. Based on local geography, climate, and industrial needs, Sichuan took the lead in establishing standards for species not yet covered by national standards—such as experimental pigs, and cats [47,48]; Hunan; meanwhile, issued local standards for experimental red crucian carp [49]. However, regardless of the province, the reviewing body is the institution’s internal IACUC, while the government (science and technology authorities) is uniformly responsible for standard formulation and post-implementation oversight, ensuring compliance with national standards while supporting the development of local experimental research.

### 2.4. Group Standards

#### 2.4.1. Group Standard for Laboratory Animal—Guideline for the Welfare of SPF Chicken T/CALAS 83-2020

The “Ethical Guidelines for the Welfare of SPF Chickens Used as Laboratory Animals” (hereinafter referred to as the SPF Chicken Welfare Standard) was published by the Chinese Association for Laboratory Animal Science in 2020. As an industry standard specific to a single animal species, it aims to standardize the management of SPF chickens, improve their quality, and better serve scientific research and production activities. The standard specifies requirements down to precise numerical values; for example, it stipulates that the floor area occupied by a single adult chicken in a cage should not be less than 750 cm^2^, and that the height of the cage at any point should not be less than 20 cm. This standard advances the welfare of laboratory animals from a matter of principle to enforceable operational guidelines. By specifying detailed requirements for husbandry, facilities and environment, and transport welfare, it provides a clear technical framework for local standards, significantly enhancing the standard’s practicality. At the same time, the standard pioneers the advanced concept of “mutual reinforcement between welfare and science”, serving as a successful model for the development of laboratory animal welfare standards as a whole [50].

#### 2.4.2. Work Specifications for Laboratory Animal Welfare and Ethics Committees T/CALAS 73-2019

The “Working Standards for Laboratory Animal Welfare and Ethics Committees” and its accompanying “Implementation Guidelines”, released by the Chinese Association for Laboratory Animal Science in 2019, represent the first specialized standards in China addressing the organizational structure and operational management of welfare and ethics committees. For the first time, these standards clearly define the proportional composition of committee members (e.g., nonlaboratory animal specialists must constitute no less than one-third of the membership), as well as term limits and evaluation systems; they establish emergency review and annual reporting systems for research institutions; and they outline requirements regarding review confidentiality and recusal. This standard shifts the focus of welfare and ethics review from “what to do” to “how to do it”, significantly enhancing the standardization and enforceability of the review system [40].

#### 2.4.3. Laboratory Animal Cage Changers T/CALAS 112-2022

The “Laboratory Animal Cage Changers” standard, published in 2022, is an innovative standard that addresses animal welfare from the perspective of equipment. The standard specifies indicators such as operational noise, airflow organization, and ease of operation for animal cage-changing equipment, aiming to minimize disturbance and stress reactions in animals during cage-changing procedures while simultaneously reducing occupational health risks for operators. This standard reflects the extension of animal welfare safeguards from “management regulations” to “hardware support” [38].

### 2.5. Industry Standards

#### 2.5.1. Guidelines for the Inspection of Laboratory Animal Welfare and Occupational Health and Safety of Personnel RB/T 018-2019

The “Guidelines for the Inspection of Laboratory Animal Welfare and Occupational Health and Safety of Personnel” is the first comprehensive standard in China to treat animal welfare and occupational health and safety of personnel as equally important inspection criteria. This standard emphasizes the positive correlation between animal welfare safeguards and personnel safety—a good housing environment, standardized operating procedures, and adequate training and education can both reduce animal suffering and lower the risk of personnel exposure to biological hazards and occupational stress. The standard systematically specifies the frequency, content, methods, and corrective action requirements for inspections, incorporating welfare safeguards from “self-declaration” into a verifiable “third-party certification” framework [41].

#### 2.5.2. Guidelines for the Assessment of Humane Endpoints in Laboratory Animals RB/T 173-2018

As a key component of the certification and accreditation industry standards, the *Guidelines for the Assessment of Humane Endpoints in Laboratory Animals* is China’s first quantitative assessment standard specifically targeting humane endpoints. Its core contributions include: establishing a multidimensional scoring system based on behavioral, physiological, and clinical indicators; it specifies thresholds for determining humane endpoints across different species and experimental types; and it proposes classification and management requirements for “planned endpoints” and “unplanned endpoints.” This standard transforms humane endpoints from vague ethical requirements into operational scientific and technical specifications, providing a quantitative basis for welfare ethics reviews [43].

Its assessment system is not limited to a single indicator but comprehensively considers changes in body weight, clinical signs, behavioral manifestations, physiological indicators, and blood biochemical parameters, establishing specific thresholds for different species and types of experiments. For example, a 20% decrease in body weight, the onset of severe respiratory distress, or a loss of responsiveness to stimuli can all serve as objective criteria for triggering intervention. By using standardized scoring forms to record the animals’ condition at regular intervals, researchers can dynamically track individual health trends, thereby intervening in a timely manner before the animal is near death to avoid unnecessary suffering [51].

## 3. Synergy Among Standards Forms a Comprehensive Laboratory Animal Welfare System

### 3.1. Synergy and Complementarity Among Standards at Different Levels

Within the standard system, national standards serve as the “constitution”, responsible for establishing basic definitions and requirements related to laboratory animal welfare; they stipulate the “3Rs” principle and the Five Freedoms as core principles of welfare work, serving a general and framework-oriented function. National standards must be applicable to all industries and scenarios nationwide; the requirements they specify are broad and general, lacking specific operational guidelines, which makes implementation challenging.

Industry standards and local standards represent specialized and regional explorations within the framework of national standards, serving as a horizontal extension of the national standards. Industry standards integrate national standards into specific sectors, transforming them into detailed, actionable standards with concrete requirements. Local standards, based on regional characteristics such as climate and dominant industries, combine national standards with local features to better align with regional needs. Group standards and enterprise standards, being the standards closest to the laboratory setting, contain the most operational requirements. Group standards are formulated by specific professional associations and offer greater flexibility, allowing them to adapt rapidly to new national and international developments to meet evolving requirements. Enterprise standards, serving as internal operational guidelines, aim to quickly adapt to market demands to maximize profits while ensuring animal welfare, and are characterized by flexibility and efficiency [52].

Standards at all levels complement and synergize with one another. Higher-level standards establish fundamental principles, while lower-level standards specify detailed requirements and are responsible for concrete implementation, ensuring that national standards are effectively transmitted from the national level to every procedure involving laboratory animals. This creates a comprehensive and coordinated system of laboratory animal welfare standards.

### 3.2. Laws and Regulations Related to Laboratory Animal Standards

Most standards regarding laboratory animal welfare are voluntary in nature and require mandatory provisions through laws and regulations. At the national level, the *Regulations on the Management of Laboratory Animals* is an administrative regulation that governs the entire process of animal research, breeding, and use. The comprehensive *Law of the People’s Republic of China on Biosafety* addresses requirements such as the safety management and safe disposal of laboratory animals. At the local level, each province has enacted targeted management regulations tailored to regional conditions. These local regulations fully take into account the impact of climatic characteristics on the operation and maintenance of local experimental facilities and on biosafety management. For example, Hainan Province’s hot and humid climate imposes higher demands on facility moisture control, air conditioning system capacity, and the prevention and control of tropical diseases; in Hunan Province, where temperature fluctuations are significant during the winter and spring seasons, priority must be given to addressing cold stress and preventing respiratory diseases [53,54].

In addition, policy documents such as the Guiding Opinions provide a reference for the issuance of standards. Laws cite standards, thereby conferring a mandatory nature upon them. For instance, Article 17 of the “Regulations on the Management” stipulates: “Units and individuals engaged in laboratory animal work shall safeguard animal welfare...” [55]. Safeguarding animal welfare is mandatory, and since the standards specify how this is to be achieved, the standards themselves become mandatory. Standards facilitate the implementation of regulations and, through the synergistic effect of the standards system, ensure the effective enforcement of laws.

## 4. Issues and Countermeasures Regarding the Implementation and Development of Standards

### 4.1. Lack of Enforceability and Insufficient Punitive Measures

As recommended guidelines, welfare standards lack enforceability during implementation. Even though laws require compliance with animal welfare provisions during experiments, they do not specify concrete operational requirements. As the hierarchy of standards moves downward, welfare requirements lack the deterrent power of legal protection and mandatory enforcement. Welfare ethics review bodies are not sufficiently rigorous; to avoid delaying experimental progress, they often blindly approve proposals without scrutinizing experimental designs. Furthermore, when institutions violate standards or compromise animal welfare, the penalties stipulated by the standards are relatively weak and lenient [31]. Notable cases include: a domestic study submitted to *Scientific Reports* was rejected because it used chloral hydrate—a substance explicitly prohibited for animal anesthesia due to its hypnotic effects without analgesic benefits; in 2022, *PLOS ONE* retracted seven papers in a single batch, involving mouse tumor volumes as high as 4621.72 mm^3^, far exceeding the criteria for humane termination, and the authors failed to provide valid ethical approval documents; Other papers were retracted because the submitted ethical approval documents for animal welfare were noncompliant. The above cases demonstrate that the noncompliant use of anesthetics and misjudgments regarding humane endpoints are not isolated incidents, but rather systemic risks arising from inadequate review mechanisms and insufficient penalties. Typical examples include: a domestic study submitted to *Scientific Reports* was rejected because it used chloral hydrate—a substance explicitly prohibited for animal anesthesia—which has only hypnotic effects and no analgesic effects, rendering the experimental process inhumane to the animals [56]; In 2022, *PLOS ONE* retracted seven papers in a single batch; these involved mouse tumor volumes as high as 4621.72 mm^3^, far exceeding humane endpoints, and the authors failed to provide valid ethical approval documents; another paper was retracted because the submitted ethical approval documents for animal welfare were noncompliant. The above cases demonstrate that the noncompliant use of anesthetics and misjudgments regarding the humane endpoint are not isolated incidents, but rather systemic risks arising from inadequate review mechanisms and insufficient penalties.

To address these issues, improve the standard system, and advance animal welfare to a higher level, future efforts should focus on strengthening the enforcement of standards. The state should enact specific legislation on laboratory animal welfare at an appropriate stage to provide a legal basis for standard implementation. Regulatory authorities should act in accordance with laws and regulations, increase the intensity and frequency of inspections of laboratory animal personnel and institutions, impose stricter penalties, and raise the cost of noncompliance, using deterrence to reduce instances of harm to laboratory animals.

### 4.2. Staff Welfare Ethics Still Need Improvement

Researchers, particularly students, vary widely in their professional competence. When performing advanced welfare procedures—such as animal restraint, determining environmental enrichment in housing facilities, assessing humane endpoints, and performing euthanasia—they may inadvertently violate standard requirements and compromise animal welfare. A 2024 survey on animal welfare awareness among Chinese graduate students revealed that their understanding of animal welfare is insufficient, and they lack practical experience; however, students in basic medical sciences demonstrated the strongest awareness of animal welfare protection, with female students outperforming male students [57]. Veterinarians, who possess both professional skills and animal protection awareness, have not fully fulfilled their core role in ensuring animal welfare. In many institutions, the role of veterinarians leans more toward disease prevention and control and protocol development, rather than acting as implementers and supervisors of animal welfare [41].

Schools and society at large should strengthen education on welfare ethics to deeply instill animal protection concepts, gradually transforming the approach to animal welfare from passive compliance to active advocacy. Schools should establish a curriculum system for laboratory animal welfare. Programs involving laboratory animal procedures should make animal welfare courses mandatory, and incorporate welfare skills such as animal restraint, pain assessment, determination of humane criteria, and euthanasia procedures into practical training. The supervisory role of veterinarians should be leveraged, and staff must undergo practical skill assessments prior to formal experiments; only those who pass may participate. This will enhance staff awareness of animal welfare and improve their operational proficiency.

### 4.3. The Onset of Compassion Fatigue Among Laboratory Personnel

Compassion fatigue, a state of physical and psychological exhaustion resulting from prolonged exposure to the suffering of patients, animals, and others, is commonly observed among nurses, veterinarians, and laboratory technicians. Witnessing the suffering and death of animals during experiments—even when procedures and euthanasia are conducted in accordance with regulations—places an increasingly heavy emotional burden on technicians. This erodes their empathy and compassion, disrupts their daily lives, and may compromise the welfare of laboratory animals [58]. In 2014, a survey by the U.S. Centers for Disease Control and Prevention found that one in six veterinarians had experienced suicidal thoughts, and one in eleven suffered from severe psychological distress [59]. A 2022 survey of 197 small animal veterinarians in Chile on compassion fatigue revealed that 90.1% of veterinarians experienced moderate to severe compassion fatigue, and the higher their self-imposed standards, the more likely they were to experience compassion fatigue [60].

When individuals struggle to maintain high standards of ethical judgment and behavioral self-control, they tend to choose the “path of least resistance”—reducing extra effort, avoiding distressing situations, and simplifying procedures. These choices often come at the expense of animal welfare. Technicians experiencing high levels of compassion fatigue are prone to simplifying procedures such as restraint and medication administration, and tend to avoid thinking about animal suffering. They may also avoid making decisions regarding humane euthanasia, thereby prolonging the animals’ suffering. On the other hand, the occurrence of compassion fatigue leads to the departure of highly skilled laboratory animal personnel, indirectly resulting in a decline in welfare standards within the institution.

To reduce compassion fatigue among staff, regular mental health screenings and counseling services should be conducted, along with the provision of psychological support. Institutions can implement job rotation systems to prevent individuals from engaging in high-stress, high-mortality experimental procedures for extended periods, while simultaneously reducing workloads. Through organizational support and institutional design, researchers can be helped to maintain compassion and professional efficacy, thereby indirectly safeguarding animal welfare.

### 4.4. Insufficient Provision of Laboratory Equipment, Medications, and Other Supplies Related to Laboratory Animal Welfare

Outdated basic infrastructure constitutes a “hardware” factor that constrains the implementation of animal welfare. Due to financial constraints, some laboratories or production units have outdated infrastructure that fails to meet the environmental standards required for animal survival; failure to replace obsolete animal restraint equipment can easily trigger stress responses in animals. In areas such as assessing pain levels, monitoring vital signs, and conducting behavioral analysis, reliance on imported equipment—which is prohibitively expensive—prevents widespread adoption. Regarding pharmaceuticals, despite the ban on anesthetics like chloral hydrate—which are highly irritating, have poor anesthetic efficacy, and cause significant toxic side effects—some experiments still use them due to their low cost [61].

Improving hardware conditions requires multistakeholder collaboration: the state should increase funding and subsidies for research institutions and production units to drive infrastructure upgrades; simultaneously, accelerate the R&D and application of domestically produced alternative equipment and pain assessment software to reduce usage costs; and strictly phase out substandard drugs such as chloral hydrate to ensure the implementation of standards.

### 4.5. Low Transparency in Laboratory Animal Welfare

Internationally, The Basel Declaration: A Historic Document Issued in 2010 encouraged researchers to communicate openly with the public regarding the welfare of laboratory animals. European countries such as the United Kingdom, France, and Spain issued statements committing to transparent communication with the public regarding animal research. The European Association for Research on Animals (EARA) is now actively encouraging organizations to sign the ‘Transparency Agreement’; signatory organizations will adhere to the following commitments: 1. Speak with clarity about when, how and why animals are used in investigation. 2. Provide adequate information to the media and the general public about the conditions under which research using animals is carried out and the results obtained from them. 3. Develop initiatives that generate greater knowledge and understanding in society about the use of animals in scientific research. 4. Report annually on progress and share experiences. Since EARA was founded in 2014, it has further developed the concept of transparency agreements in Europe, and now worldwide, and encouraged and guided institutions towards a culture of openness and transparency about the use of research animals [62]. With the increasing ease and widespread availability of information exchange, these commitments have gradually been translated into routine public communication practices. There is debate over whether a stable, positive correlation exists between institutional transparency, public trust, and animal welfare. Some argue that transparent information disclosure and public participation mechanisms may help enhance public understanding and acceptance of institutional practices, thereby providing the conditions for external oversight to facilitate institutional improvements in animal welfare. However, whether transparency can actually drive improvements in welfare standards may depend on a variety of factors, including the institution’s internal willingness to implement such measures, supporting institutional frameworks, and the effectiveness of external feedback mechanisms [63].

In China, however, neither the public nor the scientific community is typically aware of how the welfare and ethical review process for a research project is conducted. During the review process, details such as committee members’ opinions and protocol revisions are not disclosed, resulting in a lack of transparency in the work of committees overseeing animal welfare implementation. This prevents public oversight. Upon completion of a research project, information regarding the animals’ pain, suffering, and duration of procedures is rarely made public, making it impossible for outsiders to determine whether the experiment adhered to basic welfare principles.

China should establish a unified platform for disclosing information on laboratory animal welfare. Institutions should be required to disclose information related to animal welfare during experiments—provided such information is not classified—and be subject to public oversight. At the same time, penalties for violations should be specified in greater detail to eliminate formalism and effectively enhance the deterrent effect of welfare standards.

## 5. Conclusions

On the one hand, public awareness of animal welfare is growing; on the other hand, the scientific shortcomings of animal experimentation point to advanced nonanimal methods as the future direction of research. In fact, the most effective way to spare animals from suffering is to use experimental methods efficiently (the “replacement” principle of the 3Rs)—thereby reducing the production of laboratory animals—and to utilize nonliving experimental subjects [64,65]. After decades of development, China’s standards for laboratory animal welfare have made a historic leap from nonexistence to existence, and from a fragmented to a systematic framework. The various national, local, industry, and association standards issued in accordance with the “Guiding Opinions” and “Administrative Regulations” have established a multitiered, synergistic, and complementary standard system for animal welfare protection, centered on the “3Rs” principle and the Five Freedoms. The release of key standards that align with contemporary and international requirements, such as the “General Principles of Animal Welfare”, marks China’s transition from prioritizing the quality of laboratory animals to a new phase of actively ensuring the fulfillment of animals’ health and psychological needs. However, the future development of the standard system remains a long and arduous journey.

The orderly operation of the standards system requires not only sound technical specifications and institutional design, but also relies on the continuous improvement of the ethical standards and professional competence of researchers, veterinarians, and laboratory animal personnel. Currently, China still faces numerous challenges in the “last mile” of standards implementation: issues such as the lack of binding force in recommended standards, ethical reviews that are merely a formality, inconsistent awareness of animal welfare among practitioners, and the distortion of welfare standards due to occupational burnout remain prominent. While refining the text of standards is certainly important, without supporting enforcement mechanisms and staff training, even the most comprehensive standards will remain mere words on paper.

The pursuit of animal welfare is not only an expression of compassion toward laboratory animals—our fellow creatures—but also a manifestation of the responsibility and respect we bear toward scientific experimentation. Scientific progress goes hand in hand with theoretical and ethical progress; only by combining the rigid constraints of the standards system with the internalized awareness fostered through education and training—and by transforming external regulatory pressure into the industry’s intrinsic motivation—can we truly achieve high-quality, humane scientific experimentation and ensure that China’s laboratory animal welfare efforts proceed steadily and far along the path of standardization and internationalization.

## Figures and Tables

**Table 1:** Laboratory animal welfare standards.

Standard Category	Standard Name	Standard No.	Issuing Authority
National Standard	Laboratory animal—Guideline for ethical review of animal welfare	GB/T 35892-2018 [32]	State Administration for Market Regulation
	Laboratory animals—General code of animal welfare	GB/T 42011-2022 [33]	State Administration for Market Regulation
	Biological evaluation of medical devices— Part 2: Animal welfare requirements	GB/T 16886.2-2011 [34]	National Medical Products Administration
	Laboratory Animal—Guidelines for euthanasia	GB/T 39760-2021 [35]	State Administration for Market Regulation
Local Standard	Laboratory Animals—Standards of Welfare and Ethics	DB32/T 2911-2016 [36]	Jiangsu Provincial Quality and Technical Supervision Bureau
	Technical specification of ethical review for laboratory animal welfare	DB11/T 1734-2020 [37]	Beijing Market Supervision and Administration Bureau
Group Standard	Laboratory animal—Cage exchanging stage	T/CALAS 112-2022 [38]	Chinese Association for Laboratory Animal Science (CALAS)
	Group Standard for Laboratory Animal—Guideline for the Welfare of SPF Chicken	T/CALAS 83-2020 [39]	
	Laboratory animals—Work specification of the welfare and ethics committee	T/CALAS 73-2019 [40]	
	Implementation Guide for “ Laboratory animals—Work specification of the welfare and ethics committee”	T/CALAS 73-2019	
Industry Standard	Guide to inspection of laboratory animal welfare and personnel occupational health and safety	RB/T 018-2019 [41]	China National Certification and Accreditation Administration
	Protocol of animal welfare in the process of breeding, transportation and experiment of experimental animal	SN/T 3986-2014 [42]	General Administration of Quality Supervision, Inspection and Quarantine
	Guideline of assessment for humane endpoints in animal experiment	RB/T 173-2018 [43]	China National Certification and Accreditation Administration
Enterprise Standard	Ethical standards for animal experiments	Q/XWDL 1043S-2025 [44]	Xi’an Micro-Power Health Management Co., Ltd.

## Data Availability

No new data were generated or analyzed in this study. Data sharing is not applicable to this article.
